# Case Report: Co-occurrence of tubulitis and SARS-CoV-2 specific T-cells in a kidney transplant recipient

**DOI:** 10.3389/frtra.2025.1537656

**Published:** 2025-04-24

**Authors:** Ulrik Stervbo, Maximilian Seidel, Julian Uszkoreit, Sviatlana Kaliszczyk, Moritz Anft, Martin Eisenacher, Timm H. Westhoff, Nina Babel

**Affiliations:** ^1^ Center for Translational Medicine and Immune Diagnostics Laboratory, Medical Department I, Marien Hospital Herne, University Hospital of the Ruhr-University Bochum, Herne, Germany; ^2^ Berlin Institute of Health, Berlin-Brandenburg Center for Regenerative Therapies, and Institute of Medical Immunology, Charité – Universitätsmedizin Berlin, Corporate Member of Freie Universität Berlin, Humboldt-Universität zu Berlin, Berlin, Germany; ^3^ CUBiMed.RUB, Medical Faculty, Ruhr University Bochum, Bochum, Germany; ^4^ Medical Bioinformatics, Medical Faculty, Ruhr University Bochum, Bochum, Germany; ^5^ Medizinisches Proteom-Center, Medical Faculty, Ruhr University Bochum, Bochum, Germany

**Keywords:** case report, TCR-seq, COVID-19, kidney transplant, cross-reactive T cells

## Abstract

**Background:**

Kidney transplantation is associated with an increased risk of severe COVID-19 disease. Additionally, cells of the kidney express ACE-2 making them a potential target of the SARS-CoV-2 virus. Both uncontrolled viral replication and T-cell receptor (TCR) mediated cellular immunity towards the infected cells could lead to tissue destruction in the kidney. In cases where pathological findings are not always capable of providing definitive diagnosis, insights into the TCR repertoire could offer valuable information. Here we present a case of potentially infection driven tubulitis in a kidney transplant patient.

**Methods:**

The source of kidney infiltrating T-cells was assessed through next generation TCR sequencing. Using cells from the living donor and overlapping peptide pool of SARS-CoV-2 S-, N-, and M-protein (Wuhan variant), antigen specific T-cells were isolated from peripheral blood by overnight stimulation and subsequent isolation using antibodies and magnetic beads against CD154 and CD137. The clonotypes of these two samples were compared to the clonotypes in a single kidney biopsy cylinder.

**Results:**

We found that 11.1% of the repertoire of the kidney infiltrating T cells were identical to SARS-CoV-2 specific T-cells in the periphery, and only 3.1% of the repertoire was identical to allo-specific TCRs. We also observed substantial overlap between the TCR repertoires of virus-specific and donor-specific T cells, with high similarity and even identical TCR sequences present in both populations. The TCRs with dual specificity constituted a larger proportion of the allo-specific than the virus specific population. These results indicate that SARS-CoV-2 specific T-cells may directly spill into an allo-specific T cell response and that either class of T-cells may cause the observed tubulitis.

**Conclusion:**

TCR-seq of whole biopsies is a method to evaluate the ingragraft TCR repertoire can complement routine pathology and provide further insights into the mechanisms underlying a diagnosis.

## Introduction

Transplant recipients require life-long immune suppression therapy to prevent organ rejection. However, this suppression of the immune system can lead to increased risk of infections.

During the first wave of COVID-19, solid organ transplant recipients were observed to have a COVID-19 associated hospitalization rate of up to 90% and a mortality rate of 20% (1). These hospitalization and mortality rates strongly depend on the immunosuppressive regimens (2). In particular steroids and mycophenolic acid are associated with a higher hospitalization rate in kidney transplant recipients (2).

Managing viral infections in transplant recipients poses significant challenges due to their compromised immune system. In some cases, distinguishing between symptoms caused by viral infections and those related to allograft rejection can be difficult, as both conditions may present with similar clinical signs (3). T cells play an essential role in the immune system and are triggered by the recognition of cognate antigens by the T cell receptor (TCR), which can be pathogenic or allo, presented by antigen-presenting cells in the context of the major histocompatibility complex (MHC) (4).

The heterodimeric TCR is uniquely expressed on the surface of T cells with the alpha-beta configuration constituting many circulating T cells. There is an extraordinarily large variation in TCRs, which is facilitated by its variable region, which comprises three complimentary determining regions (CDR1, CDR2, CDR3). The genome encoded CDR1 and CDR2 interact with MHC molecules and stabilize the CDR3 region, which is highly polymorphic and serves as the primary site for antigen recognition (5). In fact, the highly polymorphic CDR3 region may serve as fingerprints for T cell specificity towards viral or allogeneic antigens (6). The ability to characterize the TCR repertoire and identify antigen-specific T cell clones could provide a more personalized approach to create a differential diagnose of viral vs. allo associate tissue damage.

In this case report, we utilize T-cell receptor sequencing to assess the aetiology of acute kidney transplant failure in a COVID-19 kidney transplant patient during the first wave of the SARS-CoV-2 pandemic. The patient had a 0-0-0 HLA-mismatch to the sibling who donated the kidney. We found mostly SARS-CoV-2 and few allo-specific TCRs in kidney. There was a degree of overlap between SARS-CoV-2 and allo-specific TCRs. These clonotypes had a higher frequency within the allo-specific population. We hypothesise, that SARS-CoV-2 may raise an autoimmune response with higher avidity for self than for the pathogen.

## Case description

**Figure 1:**
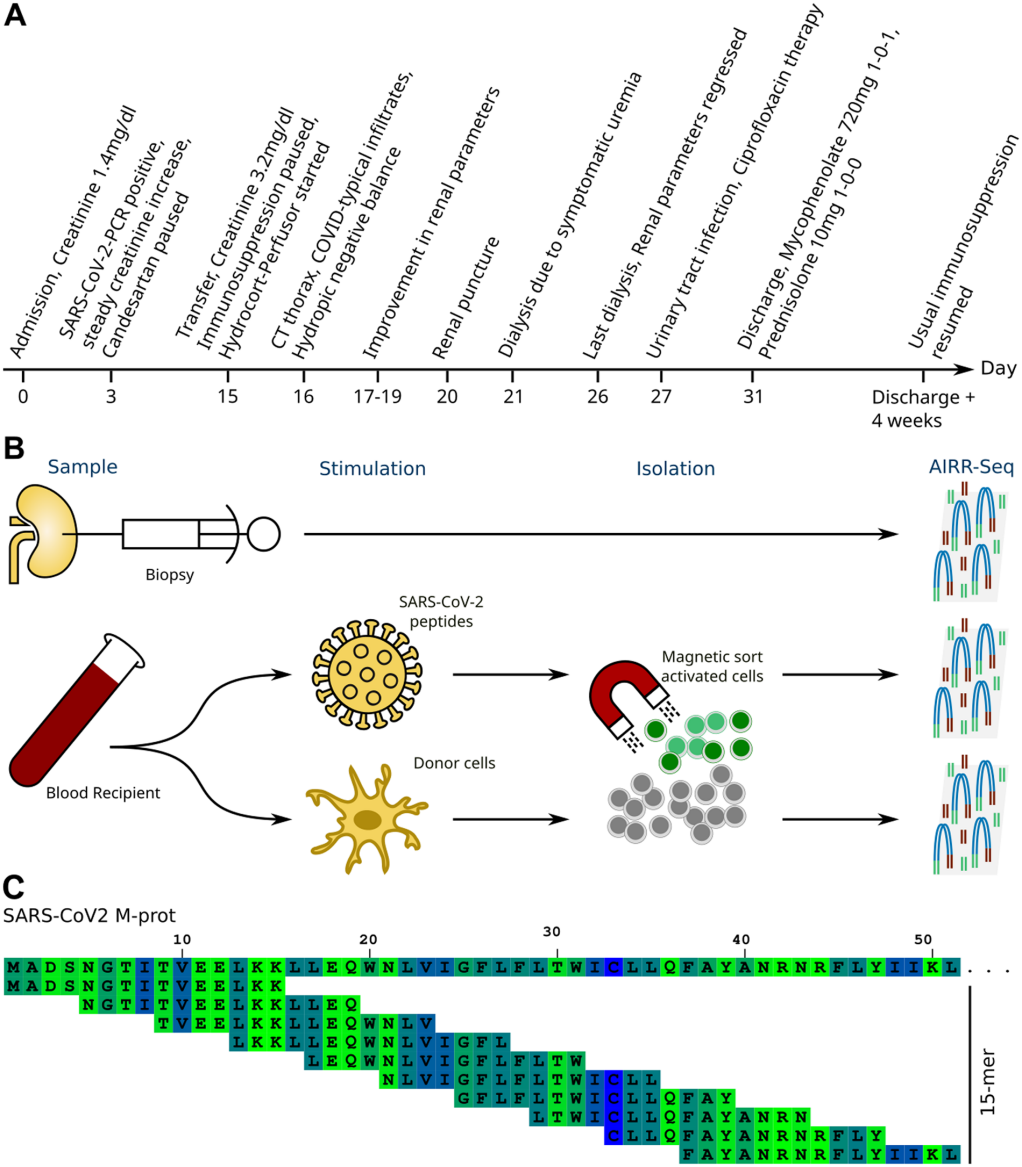
Background and assay. **(A)** Case timeline. **(B)** Schematic representation of the experimental assay to obtain TCR sequences for comparison and evaluation. **(C)** Principle of 15-mer peptides with a 11-mer overlap. The first 50 amino acids of the SARS-CoV-2 M-protein is used as example sequence.

A 56-year-old male of European descent was transferred to us with acute kidney injury from a neighbouring hospital in Germany in January 2021. The individual had undergone a kidney transplant from a living sibling 11 years prior, with a 0-0-0 HLA-mismatch. The patient was initially admitted due to COVID-19 associated dyspnoea and was transferred to our hospital 2 weeks after admission due to acute allograft failure with creatinine rise from 1.4 mg/dl (MDRD-eGFR 56 ml/min/m^2^) to 3.2 mg/dl (MDRD eGFR 16 ml/min) corresponding to AKIN 2 (7) (Figure 1A).

## Diagnostic assessment

The immunosuppressive regimen was adjusted in response to the SARS-CoV-2 infection. The agents were reduced from prednisolone, mycophenolate, and cyclosporine to 200 mg of hydrocortisone, which was infused via a syringe pump continuously over 24 h. Furthermore, we discontinued an established sequential nephron blockade with chlorthalidone and torasemide. RAS-acting agents were terminated prior to the transfer. Since these measures did not lead to a significant reduction of renal retention parameters, the aetiology of the acute kidney injury remained unclear. We have previously shown that analysis of infiltrating T cells by their adaptive immune receptor repertoire (AIRR) is useful in diagnostic specificity in case of unclear graft function deterioration (3, 7). Therefore, we obtained two biopsies—one for histopathological evaluation and one for TCR profiling. In the biopsy for histological evaluation, there was no evidence of SARS-CoV-2 virions, as assessed by *in situ* hybridization. Histology and electron microscopy showed minor mesangial IgA nephropathy with signs of tubular necrosis. Due to a progressive and symptomatic increase in the blood urea nitrogen concentration, the patient received intermittent haemodialysis for 3 days, after which the renal retention parameters declined, and renal replacement therapy was no longer required. At this time, we also received the AIRR data, which was no longer required for diagnosis as we interpreted the acute kidney injury initially as caused by tubular necrosis due to infection-associated hypotension. Four weeks after discharge, the creatinine had returned to baseline and the original immunosuppressive therapy was continued.

Since there were no detectable levels of SARS-CoV-2 virions by *in situ* hybridization, we asked if the tubulitis could have been caused by a spillover of SARS-CoV-2 specific T-cells or if there was an allo component as bystander effect to the initial infection. To this end, we utilized the obtained AIRR data.

Each T cell clone is equipped with a unique TCR which can further be used to track and infer properties of interesting T-cells (6). Each TCR has specific specificity, and only T cells with a matching antigen specific TCR proliferate during an immune reaction. By comparing antigen-specific cells isolated from one sample to another, we can gain insights into the immune cells in the latter. To pinpoint TCR sequences that are specific for SARS-CoV-2, we isolated peripheral blood mononuclear cells (PBMCs) from a blood sample (20 ml of blood). The PBMCs were stimulated overnight with a single overlapping peptide pool from SARS-CoV-2 S-, N-, and M-proteins or donor cell. The peptide pools were composed of 15-mers that cover the entire SARS-CoV-2 S-, N-, and M-proteins with an 11 amino acid overlap (Figure 1D). The cell lysate was created by depleting CD3+ T cells from PBMCs from the living donor by MACS and subjecting the CD3 free population to three rounds of snap freeze/thaw. Following this stimulation, antigen-specific T cells were isolated using magnetic beads with antibodies against the T cell activation markers CD154. SARS-CoV-2- or -specific T cells enriched in this manner underwent next-generation sequencing of the TCRβ chain as previously described (8). A biopsy was taken concurrently with the blood sample, and by comparing the clonotype repertoire obtained from SARS-CoV-2-specific or allo-specific T cells (*n* = 5,372,344 and *n* = 324,187) to the repertoire derived from the kidney biopsy (*n* = 4,181,656), the reactivity of the infiltrating T cells can be determined.

**Figure 2:**
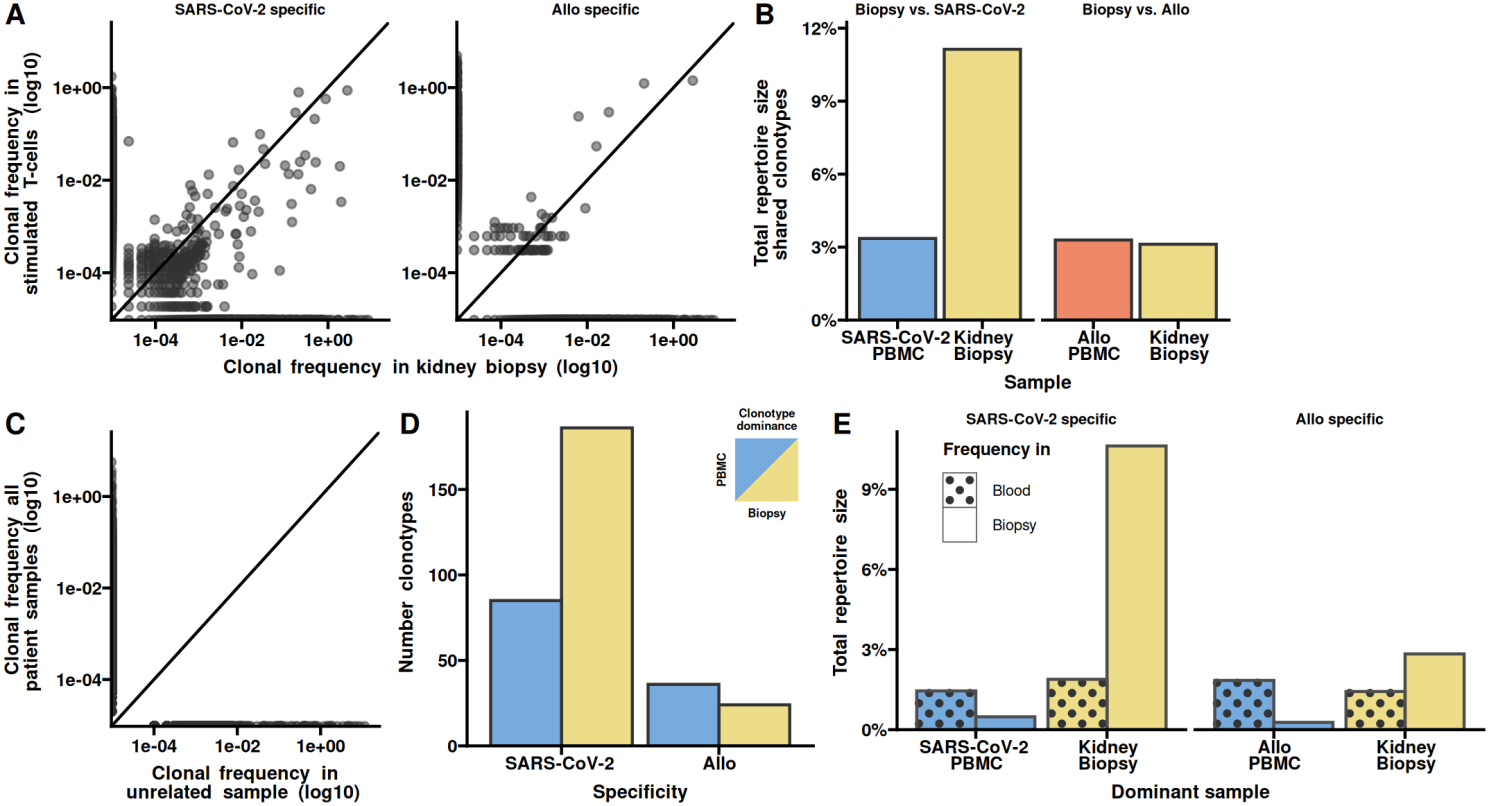
SARS-CoV-2 specific clonotypes dominate in the kidney biopsy. **(A)** Clonal frequency in kidney biopsy vs. blood of SARS-CoV-2 (left) and allo-specific TCRs (right). Each clonotype is depicted as a grey dot. The line indicates equal frequencies in both sample types (the slope is 1). **(B)** Total repertoire sizes shared between the kidney biopsy and SARS-CoV-2 or allo stimulated PBMCs. **(C)** Clonal frequency of all TCRs from the kidney biopsy and the SARS-CoV-2-specific and allo-specific samples vs. an unrelated sample from the same sequencing run. Each clonotype is depicted as a grey dot. The line indicates equal frequencies in both sample types (the slope is 1). **(D)** The number of clonotypes with higher frequency in either kidney biopsy or blood for the SARS-CoV-2- and allo-specific TCRs. The clonotypes are defined as dominant based on their frequency, so that clonotypes below the diagonal in **A** are defined to dominated in the kidney and clonotypes above the diagonal dominate in the peripheral blood. **(E)** Comparison of the total frequency of the clonotypes in either blood or biopsy samples, depending on the dominant sample and stimulation. Dominance is based on whether the clonotypes are found in larger frequencies in the kidney biopsy or blood.

We found that clonotypes in T-cells specific to SARS-CoV-2 had a larger frequency in the kidney (Figure 2A,B). In contrast, allo-specific TCRs were found in slightly higher frequencies in blood compared to the biopsy. When comparing all obtained clonotypes to the clonotypes in an unrelated TCR sample from the same sequencing run, we found no overlap, showing that the overlap to the biopsy is specific (Figure 2C). The scatterplot comparing antigen specific clonotypes with biopsy clonotypes, showed distinct patterns. SARS-CoV-2 specific TCRs had a higher frequency in the biopsy than in the blood (lower quadrants), while the allo-specific TCRs displayed the inverse (upper quadrants). To further investigate this, we evaluated the frequencies in the lower and upper scatterplot quadrants (Figure 2D). We found a larger number of TCRs specific for SARS-CoV2 in the kidney, compared to the blood, and a slightly lower number of allo-specific TCRs in the biopsy compared to the blood. Looking at the total frequency of these clonotypes we found a strong difference between biopsy and blood (Figure 2E). Taken together, the results show that SARS-CoV-2 specific clonotypes dominate in the kidney biopsy.

**Figure 3:**
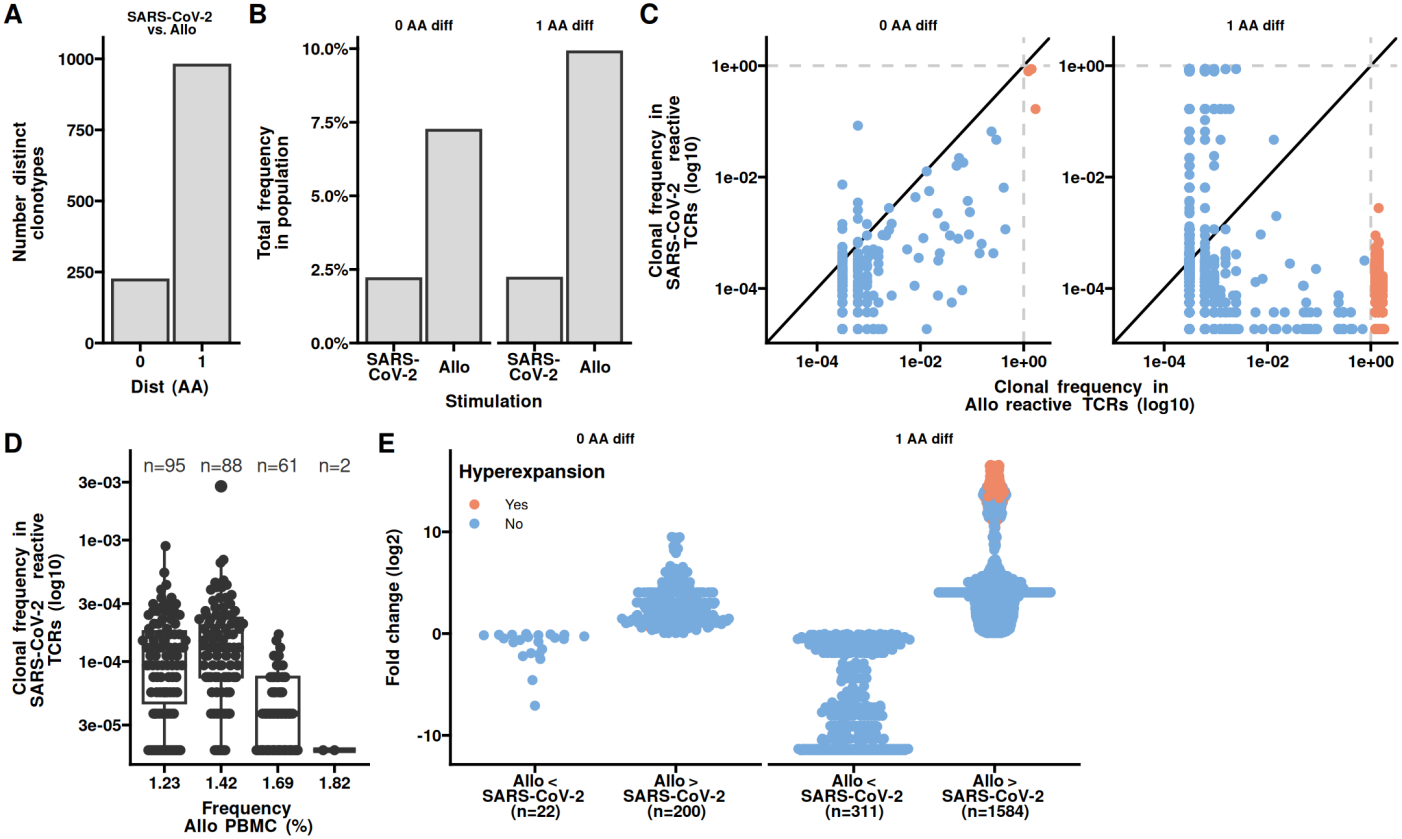
Clonotypes with both allo and SARS-CoV-2 reactivity have higher frequencies towards allo. **(A)** The number of shared clonotypes between PBMCs stimulated with SARS-CoV-2 overlapping peptide pools from S-, N-, and M-proteins or allo stimulation. Shared is defined as having an amino acid difference of 0 or 1. **(B)** The total repertoire sizes as percentages, shared under SARS-CoV-2 and allo stimulations. **(C)** Scatter plot depicting the clonotypic frequency in SARS-CoV-2 specific TCRs vs. allo-specific TCRs. Each clonotype is represented as a dot, diagonal line represents equal frequency. The dotted lines indicate hyperexpansion limit (clonal frequency ≥ 1%). **(D)** The clonal frequency of hyperexpanded SARS-CoV-2 specific TCRs in allo PBMCs. **(E)** The fold change in clonotypic expansion of shared clonotypes between PBMCs stimulated with SARS-CoV-2 overlapping peptide pools or allo.

We next analysed nucleotide sequence similarities between the SARS-CoV2 specific and allo-specific clonotypes. To this end, we calculated the hamming distance between all clonotypes as the number of different amino acids of the CDR3. An amino acid difference of 1 is generally considered similar (9). We found several identical and larger number of similar CDR3s (Figure 3A). The total frequency of identical CDR3s were similar between the two populations, while the similar clonotypes had a higher total frequency in the allo-specific population (Figure 3B). Comparing the frequency of individual clonotypes we found that clonotypes with identical and similar CDR3s had a higher frequency in the allo-specific population compared to the SARS-CoV2 population (Figure 3C). Some of these clonotypes were even hyperexpanded, that is their frequency in the population was above 1%. As similarity is a one-to-many relation, we noted a large number of SARS-CoV2 clonotypes similar to the allo-specific clonotypes (Figure 3D). When evaluating the fold-change in the identical or similar clonotypes in the SARS-CoV2 specific and allo-specific populations, we see a marked skew towards the allo-specific population (Figure 3E). Collectively, the higher frequency of clonotypes with both allo and SARS-CoV-2 specificity in the allo-specific populations indicate a higher avidity towards allo peptides.

To assess if the clonotypes identified as all or SARS-CoV2 specific could have other known specificities, we queried the curated VDJdb (10). However, none of the clonotypes were found in VDJdb. We therefore asked if the clonotypes could be termed public. To this end we queried the AIRR Data Commons (11, 12). 48 clonotypes could be identified in two other studies namely ImmuneCODE-COVID (13) and the CMV study be Emerson et al. (14). Interestingly, the clonotypes with Allo specificity only, were found in most samples of the two studies, and clonotypes with SARS-CoV2 specificity were hardly public (Table 1).

**Table 1:** Publicity of clonotypes with both allo and SARS-CoV-2 reactivity.

Study	Specificy	Number of samples in study
ImmuneCODE-COVID, 2020	Allo and SARS-CoV2	119
ImmuneCODE-COVID, 2020	Allo only	240
ImmuneCODE-COVID, 2020	SARS-CoV2 only	1
Emerson et al., 2017	Allo and SARS-CoV2	82
Emerson et al., 2017	Allo only	172
Emerson et al., 2017	SARS-CoV2 only	2

**Figure 4:**
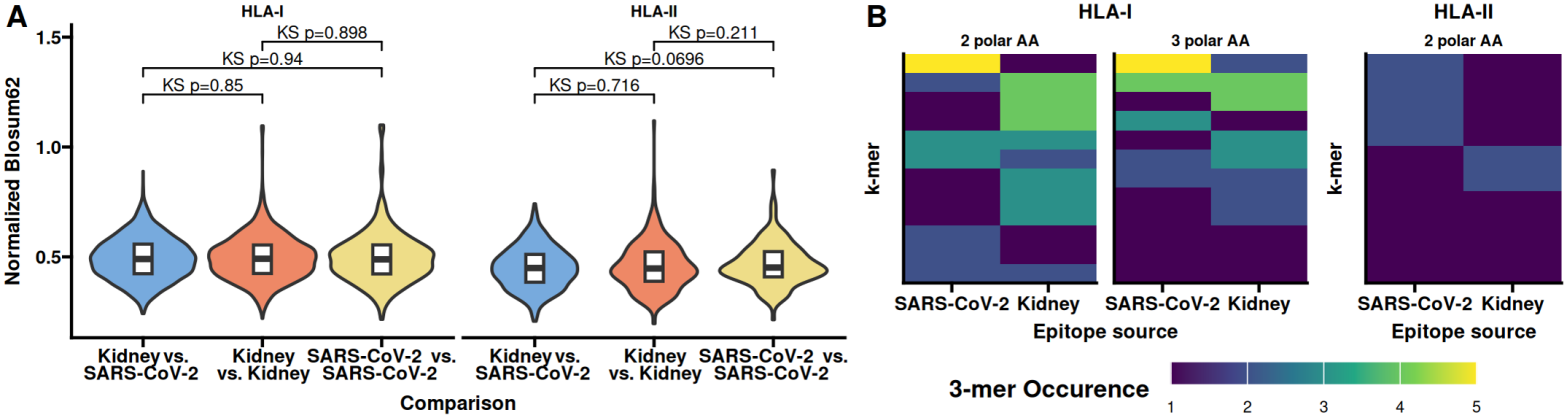
Epitope features shared between SARS-CoV-2 and kidney specific proteins occur at different frequencies. Epitopes from SARS-CoV-2 and kidney specific proteins were identified *in silico*. **(A)** Normalized Blosum62 scores from comparing epitopes between SARS-CoV-2 and kidney specific proteins, of comparing all epitopes within SARS-CoV-2 or kidney specific proteins. **(B)** Occurrence of 3-mers with 2 or 3 polar amino acids shared between SARS-CoV-2 and kidney specific HLA-I or 3-mers with 2 polar amino acids in HLA-II epitopes. No 3-mers with all polar amino acids were identified for HLA-II epitopes. Kidney specific proteins were obtained from The Human Protein Atlas. KS, Kolmogorov–Smirnov test of distributions similarity.

Similar to the similarity of the CDR3s we were wondering if epitope similarity between SARS-CoV2 and kidney specific proteins could explain the observed increased frequency of similar clonotypes in the allo-specific population. We first identified kidney specific proteins using The Human Protein Atlas and subjected these together with SARS-CoV2 S-, N-, and M-proteins to epitope prediction using NetMHCpan and NetMHCIIpan (15, 16). We utilized the Blosum62 matrix to determine the amino acid similarity between SARS-CoV-2 epitopes and those naturally occurring in the kidney. Similar epitopes are expected to be rare, so we assess the differences in distributions using the Kolmogorov–Smirnov test comparing the similarity scores between SARS-CoV-2 and Kidney epitopes to those found withing SARS-CoV-2 and Kidney, respectively (Figure 4A). We observed no significant difference in the shape or tails of the distributions. To obtain a better resolution of potential CDR3 binding sites, we reduced the epitopes to 3-mers and counted the number of polar amino acids. We found only a minor overlap in the occurrence of 3-mers with 2 or 3 polar amino acids between SARS-CoV2 and kidney protein epitopes, irrespective of HLA class (Figure 4B). These observations make it possible that there is little overlap in epitopes of SARS-CoV2 and kidney specific proteins.

## Discussion

In this care report, we presented TCR repertoire analysis of a kidney biopsy of a 56-year-old male, with a 0-0-0 HLA mismatch kidney transplant from a living sibling. We found a higher frequency of SARS-CoV-2-specific TCRs in the kidney biopsy compared to the blood, suggesting local enrichment of virus-specific T cells. Given that the kidney receives up to 25% of the cardiac output, and thereby belong to the organs with the highest blood supply in the body, it may be expected that a larger number of T-cells are captured in a biopsy (17). However, previous studies have found this number of transient T-cells to be negligible (7). The presence of T-cells in an organ biopsy largely reflects the deliberate migration of T-cells to the site of infection, where they accumulate for the duration of the infectious process (18).

It is currently not clear if COVID-19-related kidney damage is caused by viral infiltration or primarily a consequence of secondary effects such as hypoxia or shock. The dominant presence of TCRs with a SARS-CoV-2 reactivity in the kidney argues that viral infiltration is a direct cause for the functional deterioration of the transplant. The lack of SARS-CoV-2 virions, as detected by *in situ* hybridisation, appear at odds with this notion. However, it is important to note, that the TCR analysis and *in situ* hybridization were performed on two distinct cylinders. It is therefore possible that the latter biopsy did not properly sample the site of infection. T-cells, on the other hand, migrate to the inflamed kidney via chemokine receptors like CXCR3 and CXCR6, increasing the probability of sampling T-cells in a biopsy during an immune reaction in the kidney (19, 20).

Comparing the CDR3 of SARS-CoV-2- and allo-specific TCRs, we found that clonotypes that are identical or with a very high similarity, have a 3–4 times higher frequency in the allo-specific T-cells compared to the SARS-CoV-2 specific T-cells. TCR avidity is related to proliferation rate of the T-cell (21). This means, that T-cells that constitute a higher proportion may have proliferated the strongest and therefore have a higher avidity than their less proliferated counterparts. Thus, it is probable, that the TCRs with dual specificity for SARS-CoV-2 and allo, have a higher avidity for donor proteins. This indicates a chain of events, where an aberrant viral infection triggered allo-specific T-cells, with an even stronger reactivity. The promiscuity of T-cells with different avidity to different epitopes is well known. In fact, is has been demonstrated several times, that T cells with a specificity for an infectious agent like a virus, may also be allo reactive—this is also known as “heterologous immunity” (22, 23). In particular EBV and CMV are known for inducing heterologous immunity. This raises the possibility that the observed difference in avidity indeed is a common feature of the immune repertoire and unrelated to the specific epitopes evaluated here.

The probability of a meiotic recombination event in the HLA super-locus is only 1%–3% (24). Given that the donated organ is from a sibling with a 0-0-0 HLA mismatch, it is very likely that all MHC-I and all MHC-II molecules are identical (25). The allo reaction is therefore likely not due to reactions against the HLAs, but due to minor differences in the individual proteins and peptides presented on the MHC. Since not all amino acid variations in proteins are immunogenic, it is also possible that the TCRs isolated from the allo stimulation are indeed auto-specific as previously reported (26).

It is known that local tissue damage in a transplanted organ can trigger a rejection event (27). Taking the findings reported here into a broader sense, acute rejection may be caused directly by the pathogen specific T-cells. In the use analysis we could only evaluate the beta-chain of the heterodimeric T-cells receptor. Although this chain is the chain that most often interact with the peptide in the MHC, different TCR alpha chains may strongly modify the specificity (28, 29).

This study has several limitations that should be considered when interpreting our findings. As a single case report, our observations regarding TCR specificity and cross-reactivity may not be generalizable to the broader population of transplant recipients or COVID-19 patients. The technical approach, while innovative, relies on TCR sequencing and computational epitope prediction, both of which have inherent limitations and potential for error, particularly when identifying cross-reactive T cell populations. The observed overlap between SARS-CoV-2 and allo-specific TCRs warrants further investigation, as this phenomenon may be influenced by factors not fully explored in our analysis. Crucially, we did not have material to perform *in vitro* cross-reactivity validation experiments to confirm the cross-reactivity and difference in avidity of the identified TCRs. Due to the lack of material, it was also not possible to study specificity towards other infectious agents and thereby validate if the observation is specific for COVID-19 or if cross-reactivity to self is a common feature. Future studies with larger cohorts and more comprehensive validation approaches will be necessary to establish the clinical significance of our preliminary findings.

We presented a case of acute kidney injury following SARS-CoV-2 infection in a kidney transplant patient. This case illustrates how analysing the intragraft TCR repertoire can serve as a sensitive indicator of viral involvement in organ dysfunction, complementing routine pathology and providing additional insights into immune activity within the kidney (3, 7). Our findings suggest that acute tubular damage may involve immune responses not fully captured by conventional pathology, potentially driven by both virus-specific and allo-specific T cells. The cross-reactivity of T cells to viral and allo-antigens could further exacerbate these immune responses. This highlights the importance of considering the role of viral infections in transplant complications, even in the absence of detectable viral presence, and underscores the value of TCR analysis in deciphering the underlying mechanisms of organ dysfunction.

## Data Availability

The raw data supporting the conclusions of this article will be made available by the authors, without undue reservation.
